# Fatty Acid Composition of the Lipids from Atlantic Salmon—Comparison of Two Extraction Methods without Halogenated Solvents

**DOI:** 10.3390/foods10010073

**Published:** 2021-01-01

**Authors:** Jordan T. Nechev, Guro K. Edvinsen, Karl-Erik Eilertsen

**Affiliations:** The Norwegian College of Fishery Science, The Faculty of Biosciences, Fisheries and Economics, UiT The Arctic University of Norway, N-9037 Tromso, Norway; guro.k.edvinsen@uit.no (G.K.E.); karl-erik.eilertsen@uit.no (K.-E.E.)

**Keywords:** fatty acids, salmon, MTBE method, BUME method, gas chromatography

## Abstract

The scope of this paper was to apply two recently developed methods for lipid extraction: the methyl tert-butyl ether (MTBE) method and the BUME method. These two methods do not include halogenated solvents, which makes them less hazardous to the environment, less toxic, and needed in less volume compared to the standard methods for lipid extraction. Fatty acid composition of the lipids from Atlantic salmon (*Salmo salar* Linnæus, 1758) was obtained by both procedures. The methods were effective and thirty-three fatty acids were identified. The amounts of the omega-3 polyunsaturated fatty acids obtained by the MTBE method were found to be similar to the overall mean values observed in farmed salmon. The yield of the total lipids obtained by the BUME method was 13% lower. Although the methods involved different solvents, they showed similar fatty acids profile of the lipids from Atlantic salmon. Both methods were validated and some practical challenges were discussed.

## 1. Introduction

One of the Green chemistry principles states that “Auxiliary substances should be avoided wherever possible and as non-hazardous as possible when they must be used” [1]. Solvents have always been an integral part of lipid chemistry and most of them are toxic. Therefore, every step and every effort towards replacing them by alternatives with the same efficacy but with reduced toxicity must be encouraged. This paper describes our experience in comparing two recently developed methods for lipid extraction, which unlike the Folch [2] and Bligh and Dyer [3] procedures, the Golden standards in lipid chemistry: do not contain halogenated solvents.

The first alternative method was the MTBE method, which uses methanol, methyl tert-butyl ether (MTBE) and water. The method was introduced in 2008 and was shown to provide accurate and unbiased recovery of samples from bacteria, nematode, and mouse brain tissue compared to the Folch and Bligh and Dyer protocols [4]. When applied for metabolomics studies, many positive comments were reported [5,6]. MTBE was found to be a promising solvent in lipidomics [7,8,9]. Zhang, et al. [10] stated that MTBE provided a better extraction efficiency for different lipid classes. The method was reported to possess the most suitable solvent system for lipidomic studies of cell samples. Other authors ranked MTBE as one of the best solvents for lipid extraction of microalgae [11] and very effective in separation and identification of arsenolipids in algal biomass [12]. MTBE as an extractant led to higher extraction efficiency for unsaturated fatty acids, glycerophospholipids and ceramides, while methanol:chloroform favored the isolation of saturated fatty acids and plasmalogens [13]. The MTBE method was used without compromising the lipid extraction yield of lipids from European sea bass brain [14]. Furthermore, the MTBE method presented a higher extraction yield compared to the Bligh and Dyer method of sardine roe lipids. The authors also suggested, that the MTBE method could efficiently replace the Bligh and Dyer method as the traditional method for extracting lipids from marine resources [15].

The second method, the so-called BUME method, was introduced in 2012 by Löfgren et al. [16] and uses mixtures of methanol: *n*-butanol, *n*-heptane:ethyl acetate, acetic acid and water. It was developed as a fully automated method for performing total lipid extraction from plasma or serum and provided better recoveries of the acidic phospholipids than the Folch procedure. The BUME protocol showed much higher recoveries of lysophosphatidic acid. The poor recovery of this lipid class using the Folch procedure has previously been reported, and several publications suggested the use of *n*-butanol for extraction of this lipid class [16,17,18,19]. The BUME method has been applied for analyses of microalgae [20], rat plasma samples [21], nematodes [22] and urine samples [6]. The procedure was modified by Löfgren et al. [23] and the authors stated that the method was highly efficient for animal tissues.

Both methods involved solvents that are less hazardous to the environment and less toxic than halogenated solvents. Another advantage of both procedures is that the organic extract is in the upper layer and easily accessed.

The high degree of unsaturation in Atlantic salmon fats was noted in 1934 by Lovern [24]. Today this fish is an object of intense commercial and scientific interest, mainly because of its nutritional values, but also for being a sustainable source of omega-3 polyunsaturated fatty acids (PUFA) rich fishmeal and fish oil. Farmed Atlantic salmon has a slightly different lipid profile compared to wild salmon. Although the fatty acid composition of the salmon reflects the fatty acids provided in the feed [25,26], several producers now aim for producing a healthier farmed salmon with lipid content and fatty acid profiles that are closer to those in wild salmon. A total of 1,364,044 tonnes of farmed salmon was traded in 2019 in Norway [27,28]. Since salmon has become the most important fish in our region, we proposed it as a relevant subject for applying alternative and recently developed methods for lipid extraction. The emphasis was particularly to test their ability to extract omega-3 fatty acids from such an oleaginous food source.

## 2. Materials and Methods 

### 2.1. Reagents

Certified fatty acids (pentadecanoic acid (C15:0), heptadecanoic acid (C17:0), tetracosanoic acid (C24:0)) and fatty acid methyl esters (FAME) methyl pentadecanoate, methyl heptadecanoate, and methyl tetracosanoate standards were purchased from Merck KGaA, Darmstadt, Germany. Purities were guaranteed between 98 and 99.0%. Individual fatty acids were identified by comparing the retention times with known fatty acid standards: PUFA No. 1, PUFA No. 2, and PUFA No. 3 purchased from Supelco Analytical, Bellefonte, PA, USA, as well as GLC-411 (Nu-Chek Prep, Elysian, MN, USA). The other reagents were *n*-heptane ≥ 99% (Honeywell Riedel-de-Haën, Seelze, Germany), methanol Reag. Ph Eur., ACS (VWR International, Fontenay-sous-Bois, France), tert-butyl methyl ether ≥99.0% (Sigma-Aldrich, Co, St. Louis, MO, USA), 1-butanol ≥ 99.4% (Sigma-Aldrich, Co, St. Louis, MO, USA), ethyl acetate ≥ 99.5% (Sigma-Aldrich, Co, St. Louis, MO, USA), toluene ACS, ISO, Reag. Ph Eur (Merck KGaA, Darmstadt, Germany), acetic acid ≥99.8% (Honeywell Fluka, Seelze, Germany), sulfuric acid 95.0–97.0% (Honeywell Fluka, Seelze, Germany), and sodium chloride Ph Eur (VWR International BVBA, Leuven, Belgium). Distilled water was produced by a Milli-Q Academic system (Millipore SAS, Molsheim, France). FAME standard solutions were prepared by independent dilutions of aliquots from the stock solutions in *n*-heptane, and stored at −20 °C in darkness to avoid volatilization and photo degradation.

### 2.2. Sample Collection

Twenty fish of 2-year-old organic salmon of the Aquagen strain, reared at Aquafarm at Årberg, Senja Island, Troms county, Norway (69.2° N, 16.9° E), were collected in March 2018. The fish were bath anesthetized using benzocaine (30–40 mg/L), killed by gill cutting and bled out in circulating ice water for 30 min. After filleting the fish, all fillets were ground in a meat mincer, mixed, and frozen at −50 °C until analyses.

### 2.3. MTBE Method

**Figure 1:**
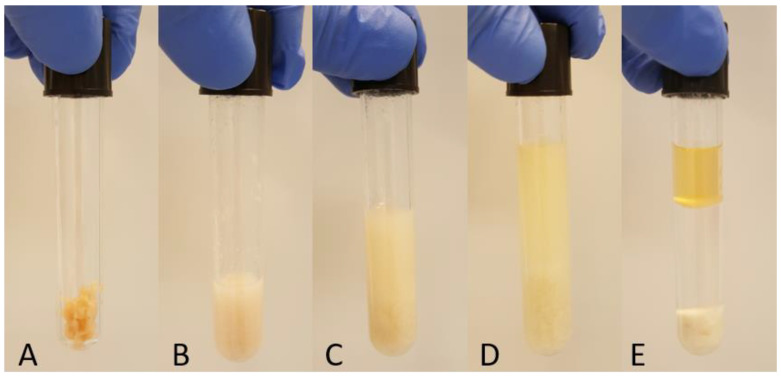
Extraction steps of the MTBE method: (**A**) One gram of salmon; (**B**) Salmon tissue + methanol; (**C**) Salmon + methanol + MTBE; (**D**) Salmon + methanol + MTBE + distilled water; (**E**) Separation after centrifugation.

The method was introduced by Matyash et al. [4] and modified by Sostare et al. [29] who reduced the volume of MTBE, resulting in methanol:MTBE:water ratio 2:2.6:2.4 *v/v/v*. To simplify the procedure, we used the solvents in a 2:2.5:2.5 ratio. One gram of defrosted ground salmon was placed in a glass tube with Teflon cap (Figure 1A) and 0.500 mL of C17:0, 10 mg/mL in methanol was added as internal standard (IS). After the addition of 1.5 mL methanol, the sample was vortexed for 1 min (Figure 1B) and 2.5 mL of MTBE was added. The glass tube was filled with nitrogen and incubated for 30 min at room temperature in a shaker (Figure 1C). Distilled water (2.5 mL) was added, vortexed and after 5 min of incubation at room temperature (Figure 1D), the sample was centrifuged at 1000× *g* for 10 min (Figure 1E). The upper (organic) phase was collected, the lower phase was re-extracted with 2 mL of MTBE, vortexed, and after 5 min of incubation at room temperature, was centrifuged again at 1000× *g* for 10 min. The upper phase was collected, combined with the first one and dried under nitrogen. The total lipid extract was dissolved in 5 mL toluene. After taking an aliquot for the methylation procedure, the vial was filled with nitrogen and stored at −20 °C.

### 2.4. BUME Method

**Figure 2:**
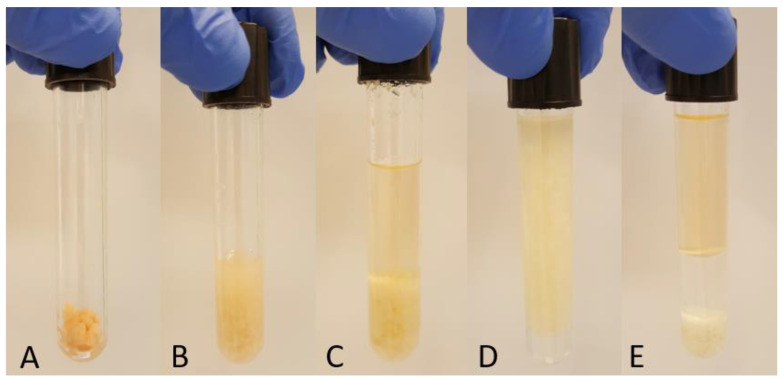
Extraction steps of the BUME method: (**A**) One gram of salmon; (**B**) Salmon tissue + butanol:methanol; (**C**) Salmon + butanol:methanol + heptane:ethyl acetate; (**D**) Salmon + butanol:methanol + heptane:ethyl acetate + acetic acid; (**E**) Separation after centrifugation.

The method was introduced by Löfgren et al. [16] and modified by Löfgren et al. [23]. One gram of ground salmon was placed in a glass tube with Teflon cap (Figure 2A) and 0.500 mL of C17:0, 10 mg/mL in methanol was added as internal standard. After adding 0.250 mL methanol, 2.250 mL of *n*-butanol was added to reach ratio *n*-butanol:methanol 3:1 and the sample was vortexed for 1 min (Figure 2B). *n*-Heptane:ethyl acetate mixture (3:1, 3 mL) was added, and the sample was vortexed for 1 min (Figure 2C). Acetic acid (1%, 3 mL) was added and after 1 min of vortexing (Figure 2D), the sample was centrifuged at 1000× *g* for 10 min (Figure 2E). The upper (organic) layer was collected, the remaining aqueous phase was re-extracted with 2 mL of *n*-heptane:ethyl acetate (3:1), vortexed for 1 min, and centrifuged at 1000× *g* for 10 min. The upper phase was collected, combined with the first one and dried under nitrogen. The total lipid extract was dissolved in 5 mL toluene. After taking an aliquot for the methylation procedure, the vial was filled with nitrogen and stored at −20 °C.

### 2.5. Fatty Acids Methyl Esters

Fatty acid methyl esters were prepared from the total lipids by acid-catalyzed transesterification, as described by Christie [30]. One per cent of the total lipid extract (50 μL) was transferred into a DURAN^®^ test tube, followed by the addition of toluene (0.950 mL) and 2 mL of 1% sulfuric acid in methanol. The sample was saturated with nitrogen and left at 50 °C overnight. Water (5 mL) containing sodium chloride (5%) was added and the required esters were extracted twice with *n*-heptane (3 and 2 mL). After 10 min on the bench, the sample was separated into two layers (without centrifugation), and the upper phase was collected with a glass Pasteur pipette. The *n*-heptane layers were combined and dried under nitrogen. The FAME obtained were resuspended in 100 μL of *n*-heptane, transferred into GC tubes and analyzed.

### 2.6. Gas Chromatographic Analysis

The fatty acid composition was determined using an Agilent 6890N gas chromatograph (Agilent Technologies, Santa Clara, CA, USA) equipped with Agilent 7683B autoinjector and flame ionization detector. Helium was the carrier gas with a flow rate of 1.6 mL/min. The individual fatty acids (FA) were separated according to their different migration rates through a Varian CP7419 capillary column (50 m × 250 μm × 0.25 μm, Agilent Technologies, Santa Clara, CA, USA). After holding the temperature at 60 °C for 1 min, the column was temperature-programmed at 30 °C/min to 130 °C, then at 1.3 °C/min to 195 °C, then at 30 °C/min to 240 °C where it was held for 10 min. The temperature of the injector was 240 °C, and the temperature of the detector was 250 °C.

## 3. Results and Discussion

### 3.1. Results

The identification of FA was based on the comparison of the retention times of standard methyl esters. The percentage of the individual FA was determined as the area of the individual FA peak in the chromatogram divided by the sum of the areas of all identified FA peaks. The amount of the various FAs was calculated from the internal standard added. Amount of FA (mg) per 1 g sample (salmon):

(1) $$Amount\;FA\;\left(\mathrm{mg}\right)=\frac{Peak\;area\;FA}{Peak\;area\;IS\;}\times \;\frac{Amount\;of\;IS\;added\;\left(\mathrm{mg}\right)}{Weight\;sample\;\left(\mathrm{mg}\right)}$$

The total lipid (TL) extract of 1 g salmon, including 5 mg of IS added before extraction, was 113.5 mg (±5.0) for the MTBE method, and 98.3 mg (±8.9) for the BUME method. Thirty-three FA were identified with overall weight 94.8 mg (82.20% of TL) for the MTBE method, and 82.4 mg (82.15% of TL) for the BUME method (Table 1).

The amounts of the omega-3 long-chain PUFA eicosapentaenoic acid (EPA; 20:5 n-3), docosapentaenoic acid (DPA; 22:5 n-3) and docosahexaenoic acid (DHA; 22:6 n-3) in salmon fillet were 0.42%, 0.16%, and 0.70% for the MTBE method. These amounts were similar to the overall mean values (0.46% EPA, 0.20% DPA, 0.74% DHA) observed in the fillet of farmed salmon collected in the same year (2018) from Southern, Mid and Northern part of Norway [31,32]. Although the amounts of omega-3 PUFA were lower for the BUME method −0.37% EPA, 0.14% DPA, and 0.61% DHA, they were still above the overall minimal values observed in farmed salmon.

The higher amount of the TL fraction for the MTBE method could be explained with the fact, as observed by Löfgren et al. [23], that the polar and negatively charged phospholipids, such as phosphatidylinositol (PI), phosphatidylglycerol (PG) and phosphatidylserine (PS), were more efficiently extracted with the MTBE method compared to both BUME and Folch methods. The authors also proposed that the MTBE fraction could extract more polar compounds [23].

In our view, the MTBE and BUME procedures appeared to be effective for the extraction of omega-3 fatty acids from fish. However, we are aware that further research is needed to compare both methods in detail with the established Golden standards in lipid chemistry—Folch and Bligh and Dyer. Such research directions could include comparison of separated lipid classes, analyzing TL fractions for polar compounds, investigating the sterol fraction, etc. It is also likely that the MTBE method shows higher efficiency than the BUME method in lean fish, such as cod.

### 3.2. Method Validation

C17:0 was used as internal standard, dissolved in methanol in concentration 10 mg/mL and 0.5 mL was added to each sample. C15:0 and C24:0 were used for spiking and validating the methods. These three fatty acids were chosen since they have been reported only in trace amounts and less than 1% of total fatty acids in salmon [33,34]. They are saturated, which makes them not susceptible to oxidation and stable in solvent. Another argument for using them is the difference in their retention times—C15:0 appears in the first part of the chromatogram while C24:0 is close to its end.

The spiking volume applied was 1 mL of the appropriate standard solution of C15:0 and C24:0 in a solvent corresponding to each method. C15:0 was dissolved in methanol (for the MTBE method) and in 1-butanol (for the BUME method). Spiking volume was added as a part of the corresponding solvent in the extraction procedure. C24:0 was dissolved at room temperature in MTBE and the spiking volume was added with the same solvent during the extraction (for MTBE method). C24:0 could not be dissolved in any of the solvents for the BUME method (methanol, *n*-butanol, ethyl acetate, and *n*-heptane) in the highest concentration for spiking −15 mg/mL. Actually, we could not obtain any exact data about its solubility in nonhalogenated solvents. We found that C24:0 dissolves easily in MTBE when warmed up to 30 °C and the solution is stable after cooling at room temperature. Therefore, spiking with C24:0 in the BUME method was performed by adding the amount for spiking, dissolved in MTBE, into an empty glass tube and evaporating the solvent under nitrogen. After adding 1 g salmon, the extraction procedure followed as described above. Four concentrations were chosen for spiking −0.06 mg/mL, 5 mg/mL, 10 mg/mL, and 15 mg/mL, representing instrumental LOQ + three equidistant calibration points. Because of the 10 times dilution during extraction and methylation procedures, the above concentrations became 6 μg/mL, 500 μg/mL, 1000 μg/mL, and 1500 μg/mL in the FAME sample, injected on GC. Standard calibration curves for C15:0 methyl ester and C24:0 methyl ester were prepared in n-heptane, using the same four concentration points.

Both methods were validated by evaluating parameters, such as linearity, limit of detection (LOD), limit of quantification (LOQ), accuracy (in terms of apparent recovery), precision (in terms of repeatability and intermediate precision), recovery, matrix effect and process efficiency. The terms “recovery” and “apparent recovery” were used as described by IUPAC recommendations [35]. Only one analyst conducted all the experiments.

#### 3.2.1. MTBE Method

Method selectivity was found to be satisfying for C15:0 and C24:0 as no interference peaks were detected. Method linearity was evaluated through the coefficient of correlation (r2) of the analytical curves at the concentration levels 0 (blank matrix), 60, 5,000, 10,000 and 15,000 μg/mL. The analytical curves presented a good linearity with r2 = 0.9995 for C15:0 and 0.9950 for C24:0. The regression line slope was 0.00206 with a standard error of 0.001. The intercept was 0.05332 with a standard error of 0.004 for C15:0. In the case of C24:0, the slope was 0.00210 with a standard error of 0.001. The intercept was 0.06470 with a standard error of 0.015. The investigation of the residual sum of squares found that all relative residuals were less than 20% and acceptable (Table 2).

The instrumental LOD and LOQ were estimated in solvent (*n*-heptane) with injecting a standard solution containing decreasing concentrations of C15:0 and C24:0. The LOD and LOQ for both fatty acids were determined to be 2 μg/mL and 6 μg/mL, representing signal-to-noise ratio of 3:1 and 10:1, respectively. We could not obtain a completely blank matrix, since C15:0 and C24:0 were present in the matrix in concentrations a little higher than instrumental LOQ. Spiking with 60 μg/mL, resulted in increasing the detector response with 35% for C15:0 and 44% for C24:0. The approach for determination of LOD and LOQ was based on the standard deviation of the blank, according to the guideline EP17 of The Clinical and Laboratory Standards Institute [36,37]. Limit of blank (LOB) was estimated by measuring replicates of a blank sample and calculating the mean result and the standard deviation (SD):

LOB = mean blank + 1.645(SD blank)

LOD was determined by utilizing both the measured LOB and test replicates of a sample known to contain a low concentration of analyte:

LOD = LOB + 1.645(SD low concentration sample)

The values calculated for LOD were 24 μg per gram salmon fillet for C15:0 and 23 μg/g for C24:0. LOQ were set to be 3 × LOD and were 73 μg/g salmon fillet for C15:0 and 70 μg/g for C24:0.

Accuracy was evaluated through recovery assays at four different concentration levels (60, 5000, 10,000 and 15,000 μg/L) and apparent recoveries were calculated (Table 2). Precision was evaluated regarding repeatability and intermediate precision by estimating the relative standard deviation (RSD) of the recovery percentage for each spiked level (Table 2). The analytical batch included four replicates that were extracted and injected once in the chromatographic system on the same day. This was repeated for each spiking level. Intermediate precision and recovery were assessed from further spike recovery assays, with two more batches (comprising two replicates each) carried out on different days over a period of three months. In general, the number of analyzed samples was 4 + 2 + 2, for each spiking concentration.

**Figure 3:**
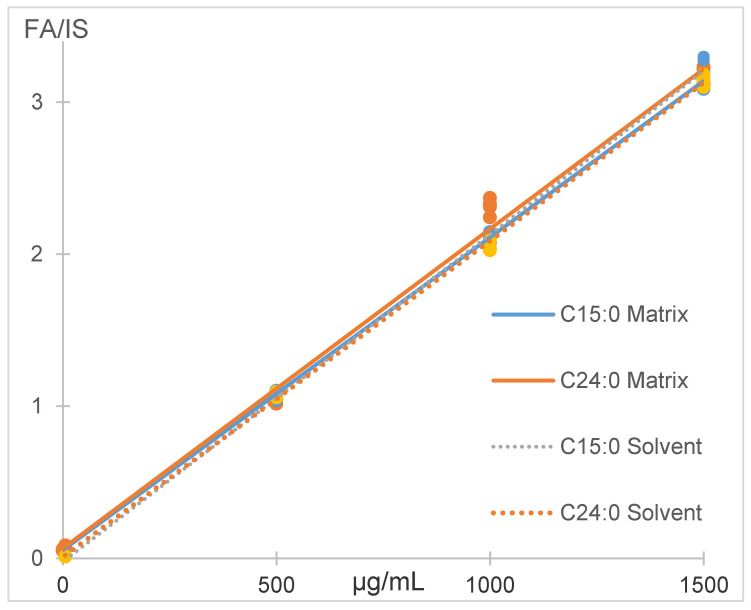
Matrix effect for C15:0 and C24:0 with the MTBE method.

The matrix effect was calculated comparing the slope of the curves prepared in solvent and in spiked matrix. For C15:0 the matrix effect was 99% and 103% for C24:0. Process efficiency, including recovery and matrix effect, was 111% for C15:0 and 126% for C24:0. The recoveries were very close to 100%, but higher only for the lowest spiking concentration, because of the analytes’ presence in matrix. Slope differences of 3% or less were found for both fatty acids investigated, in solvent and matrix, which indicates that calibration in solvent could be safely applied to quantify fatty acids in salmon (Figure 3).

#### 3.2.2. BUME Method

No interference peaks were detected for C15:0 and C24:0, indicating satisfying method selectivity. Method linearity was evaluated through the coefficient of correlation (r2) of the analytical curves at the concentration levels 0 (blank matrix), 60, 5000, 10,000 and 15,000 μg/mL. The analytical curves presented a good linearity with r2 = 0.9995 for C15:0 and 0.9984 for C24:0. The regression line slope was 0.00195 with a standard error of 0.001 for C15:0. The intercept was 0.05367 with a standard error of 0.004. In the case of C24:0, the slope was 0.00212 with a standard error of 0.001. The intercept was 0.06099 with a standard error of 0.008. The investigation of the residual sum of squares found that all relative residuals were less than 20% and acceptable (Table 3).

The instrumental LOD and LOQ were estimated to be 2 μg/mL and 6 μg/mL (See Section 3.2.1. MTBE method validation). C15:0 and C24:0 were present in the matrix and spiking with 60 μg/mL, resulted in increasing the detector response with 57% for C15:0 and 51% for C24:0. The values calculated for LOD were 26 μg per gram salmon fillet for C15:0 and 41 μg/g for C24:0. LOQ were set to be 3 × LOD and were 77 μg/g salmon fillet for C15:0 and 124 μg/g for C24:0.

Accuracy was evaluated through recovery assays at four different concentration levels (60, 5000, 10,000 and 15,000 μg/L) and apparent recoveries were calculated (Table 3). Precision was evaluated regarding repeatability and intermediate precision by estimating the RSD of the recovery percentage for each spiked level (Table 3). Intermediate precision and recovery were assessed from further spike recovery assays, following the same scheme as for the MTBE method validation.

**Figure 4:**
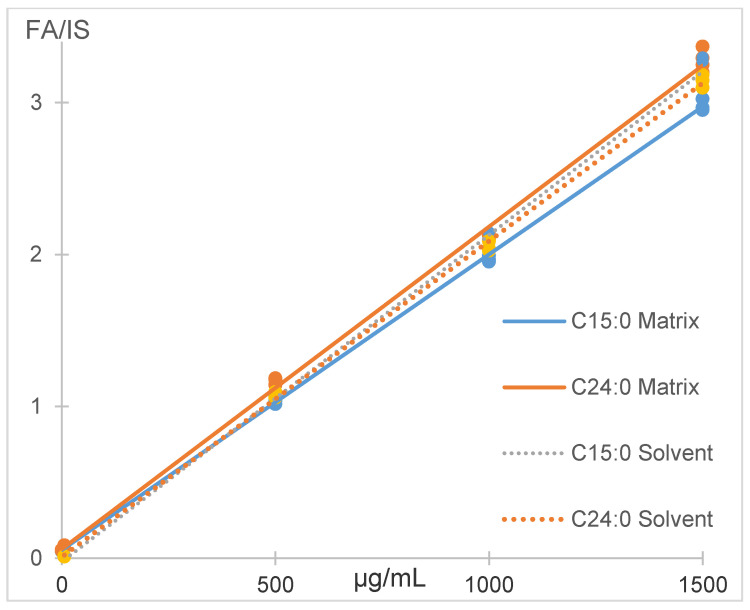
Matrix effect for C15:0 and C24:0 with the BUME method.

The matrix effect for C15:0 was 93% and 104% for C24:0. Process efficiency, including recovery and matrix effect, was 113% for C15:0 and 147% for C24:0. The slope differences were 6.5% for C15:0 and 4% for C24:0 in solvent and matrix indicating that calibration in solvent could be safely applied to quantify fatty acids in salmon (Figure 4).

Based on the insignificant matrix effect, the similarity of the slopes and the linearity obtained for both fatty acids in both methods, we can conclude with great certainty that method LOD and LOQ were little lower than calculated from the formula, and actually approaching the instrumental LOD and LOQ. That means, finally, 20 μg (LOD) and 60 μg (LOQ) per gram salmon fillet for C15:0 and C24:0, for the MTBE method, as well as for the BUME method. Another argument that supports this conclusion is that some minor fatty acids were easily detected in salmon extracts with distinct and quantifiable peaks, with acceptable RSD, in amounts around the instrumental LOQ and even less (Table 1).

## 4. Conclusions

Fatty acid composition of the total lipids from organic Atlantic salmon was obtained by the MTBE and the BUME methods, two extraction methods which do not involve any halogenated solvents. Both methods showed a similar FA profile of the lipids from Atlantic salmon. The yield of the TL obtained by the BUME method was 13% lower, whereas the proportional content of each FA was almost identical with both methods. The methods’ most significant advantages were that the applied solvents were halogen-free, and therefore less hazardous to the environment, less toxic than the halogenated ones, and less volume was needed compared to the standard procedures for lipid extraction. Another advantage of both methods is that the organic extract is in the upper layer after separation and easily accessed. Although the longer time needed for evaporation (the ether absorbs some water whereas *n*-butanol has a higher boiling point), these procedures appeared to be very effective for extraction of omega-3 fatty acids. Despite the lack of comparison in this article of both methods with the established Golden standards in lipid chemistry-Folch and Bligh and Dyer protocols, the MTBE and the BUME methods deserve more attention, and we encourage lipid scientists to use and develop them further.

## Figures and Tables

**Table 1:** Fatty acid as proportional content (% of total identified FA), and absolute amount (mg/g fish) in Atlantic salmon obtained by MTBE method and BUME method ^1^.

Fatty Acid	MTBE		BUME	
	Proportional	Absolute Content	Proportional	Absolute Content
	Content (%)	(mg/g ww)	Content (%)	(mg/g ww)
C12:0	0.05 ± 0.00	0.05 ± 0.01	0.05 ± 0.00	0.04 ± 0.00
C14:0	4.28 ± 0.03	4.05 ± 0.20	4.20 ± 0.06	3.46 ± 0.30
C14:1	0.05 ± 0.00	0.05 ± 0.00	0.05 ± 0.04	0.04 ± 0.00
C15:0	0.28 ± 0.00	0.26 ± 0.01	0.28 ± 0.01	0.23 ± 0.02
C16:0	11.88 ± 0.05	11.26 ± 0.55	11.73 ± 0.07	9.66 ± 0.83
C16:1 n-7	4.59 ± 0.01	4.35 ± 0.21	4.54 ± 0.03	3.74 ± 0.32
C17:0 ^2^	5.28 ± 0.26	5.00 ± 0.00	6.10 ± 0.48	5.00 ± 0.00
C16:2 n-4	0.33 ± 0.00	0.32 ± 0.02	0.33 ± 0.00	0.27 ± 0.02
C16:3 n-4	0.17 ± 0.00	0.16 ± 0.01	0.16 ± 0.00	0.13 ± 0.01
C18:0	2.85 ± 0.02	2.71 ± 0.14	2.81 ± 0.02	2.31 ± 0.20
C18:1 n-12	0.97 ± 0.02	0.92 ± 0.05	0.96 ± 0.02	0.79 ± 0.08
C18:1 n-9	14.89 ± 0.06	14.12 ± 0.71	14.77 ± 0.13	12.18 ± 1.09
C18:1 n-7	2.47 ± 0.00	2.34 ± 0.11	2.43 ± 0.02	2.00 ± 0.17
C18:2 n-6	12.37 ± 0.05	11.73 ± 0.58	12.25 ± 0.10	10.09 ± 0.90
C18:3 n-6	0.07 ± 0.00	0.06 ± 0.00	0.07 ± 0.00	0.06 ± 0.01
C18:3 n-4	0.12 ± 0.00	0.11 ± 0.01	0.12 ± 0.00	0.10 ± 0.01
C18:3 n-3	2.25 ± 0.01	2.13 ± 0.11	2.27 ± 0.01	1.87 ± 0.16
C20:0	0.17 ± 0.00	0.16 ± 0.01	0.17 ± 0.00	0.14 ± 0.02
C20:1 n-9	1.20 ± 0.02	1.14 ± 0.05	1.18 ± 0.01	0.98 ± 0.09
C18:4 n-3	8.30 ± 0.05	7.87 ± 0.40	8.28 ± 0.11	6.83 ± 0.64
C20:2 n-6	0.77 ± 0.01	0.73 ± 0.04	0.75 ± 0.01	0.62 ± 0.06
C20:3 n-6	0.14 ± 0.00	0.13 ± 0.01	0.14 ± 0.00	0.12 ± 0.01
C20:4 n-6	0.30 ± 0.01	0.29 ± 0.01	0.30 ± 0.00	0.25 ± 0.02
C20:3 n-3	0.09 ± 0.00	0.08 ± 0.01	0.09 ± 0.01	0.07 ± 0.01
C22:1 n-11	9.44 ± 0.05	8.96 ± 0.46	9.35 ± 0.09	7.70 ± 0.70
C22:1 n-9	2.00 ± 0.01	1.89 ± 0.09	1.98 ± 0.02	1.64 ± 0.15
C20:5 n-3	4.44 ± 0.02	4.21 ± 0.20	4.45 ± 0.03	3.66 ± 0.27
C22:2 n-6	0.07 ± 0.01	0.07 ± 0.01	0.06 ± 0.00	0.05 ± 0.01
C24:0	0.30 ± 0.00	0.29 ± 0.01	0.30 ± 0.01	0.24 ± 0.02
C24:1 n-9	0.22 ± 0.02	0.21 ± 0.02	0.20 ± 0.01	0.16 ± 0.01
C22:4 n-6	0.61 ± 0.01	0.58 ± 0.03	0.59 ± 0.01	0.49 ± 0.04
C22:5 n-3	1.71 ± 0.00	1.62 ± 0.07	1.70 ± 0.00	1.40 ± 0.11
C22:6 n-3	7.35 ± 0.04	6.97 ± 0.29	7.37 ± 0.12	6.06 ± 0.39
Total	100.00 ± 0.02	94.82 ± 4.39	100.00 ± 0.00	82.38 ± 6.64

^1^ Values are expressed as mean ± SD from four parallel extractions and GC-analyses. ^2^ Corresponding to 5 mg IS added in all samples.

**Table 2:** Relative residuals, accuracy, precision and recovery obtained with the MTBE method ^1^.

	Relative Residuals %	Apparent Recovery (*n* = 4)	Repeatability RSD% (*n* = 4)	Recovery (*n* = 8)	Intermediate Precision RSD% (*n* = 8)
**C15:0**					
6	5.2 to 12.4	148 (122–174)	11	144 (122–166)	18
500	−3.3 to 1.8	99 (96–103)	2	103 (101–104)	2
1000	−1.7 to 1.6	100 (98–102)	1	99 (95–103)	5
1500	−1.7 to 2.4	100 (97–103)	2	103 (100–105)	3
**C24:0**					
6	4.3 to 9.4	143 (119–167)	10	168 (125–212)	31
500	−5.0 to −9.0	93 (90–96)	2	107 (99–115)	9
1000	3.5 to 9.4	107 (103–111)	2	107 (101–112)	6
1500	−3.6 to 0.5	98 (95–101)	2	105 (102–108)	3

^1^ Mean values and 95% confidence intervals (in parentheses).

**Table 3:** Relative residuals, accuracy, precision and recovery obtained with the BUME method ^1^.

	Relative Residuals %	Apparent Recovery (*n* = 4)	Repeatibility RSD% (*n* = 4)	Recovery (*n* = 8)	Intermediate Precision RSD% (*n* = 8)
**C15:0**					
6	9.3 to 17.2	181 (150–212)	11	197 (151–242)	28
500	−1.2 to 3.9	101 (98–105)	2	97 (95–100)	3
1000	−0.4 to −2.4	98 (97–100)	1	95 (94–96)	2
1500	−0.8 to 1.7	101 (99–103)	1	96 (94–98)	3
**C24:0**					
6	2.6 to 14.9	144 (91–197)	23	256 (233–279)	11
500	1.6 to 5.6	104 (100–107)	2	104 (98–111)	8
1000	−2.4 to −3.8	97 (96–98)	1	102 (99–104)	3
1500	−1.4 to 3.8	101 (97–105)	2	102 (96–107)	6

^1^ Mean values and 95% confidence intervals (in parentheses).

## Data Availability

The data presented in this study are available on request from the corresponding author.
